# Transient incubation of cultured hippocampal neurons in the absence of magnesium induces rhythmic and synchronized epileptiform-like activity

**DOI:** 10.1038/s41598-021-90486-y

**Published:** 2021-05-31

**Authors:** Miranda Mele, Ricardo Vieira, Bárbara Correia, Pasqualino De Luca, Filipe V. Duarte, Paulo S. Pinheiro, Carlos B. Duarte

**Affiliations:** 1 CNC-Center for Neuroscience and Cell Biology, Faculty of Medicine, University of Coimbra, Rua Larga, 3004-504 Coimbra, Portugal; 2 Institute for Interdisciplinary Research, Coimbra, Portugal; 3 Department of Life Sciences, University of Coimbra, Coimbra, Portugal

**Keywords:** Neuroscience, Cellular neuroscience, Diseases of the nervous system

## Abstract

Cell culture models are important tools to study epileptogenesis mechanisms. The aim of this work was to characterize the spontaneous and synchronized rhythmic activity developed by cultured hippocampal neurons after transient incubation in zero Mg^2+^ to model *Status Epilepticus*. Cultured hippocampal neurons were transiently incubated with a Mg^2+^-free solution and the activity of neuronal networks was evaluated using single cell calcium imaging and whole-cell current clamp recordings. Here we report the development of synchronized and spontaneous [Ca^2+^]_i_ transients in cultured hippocampal neurons immediately after transient incubation in a Mg^2+^-free solution. Spontaneous and synchronous [Ca^2+^]_i_ oscillations were observed when the cells were then incubated in the presence of Mg^2+^. Functional studies also showed that transient incubation in Mg^2+^-free medium induces neuronal rhythmic burst activity that was prevented by antagonists of glutamate receptors. In conclusion, we report the development of epileptiform-like activity, characterized by spontaneous and synchronized discharges, in cultured hippocampal neurons transiently incubated in the absence of Mg^2+^. This model will allow studying synaptic alterations contributing to the hyperexcitability that underlies the development of seizures and will be useful in pharmacological studies for testing new drugs for the treatment of epilepsy.

## Introduction

Epilepsy is a neurological disorder characterized by recurrent unprovoked seizures, with multiple possible causes and highly diversified symptoms. The disease affects approximately 65 million people worldwide, from all ages and genders^1–3^. The study of the mechanisms involved in the genesis and propagation of epileptic seizures has been a challenge in the field of neuroscience for many decades. Despite the advances in the understanding of epileptogenesis, the mechanisms underlying the development of seizures are poorly understood.

Since human epilepsies display a wide range of behavioural and electrical manifestations, it is not surprising that a large number of animal and in vitro models have been used to study this disease. The available in vivo and in vitro epilepsy models commonly use chemical or direct electrical stimulation. Typically, these models are characterized by an unbalance in the excitatory/inhibitory equilibrium, due to an enhancement of the activity of excitatory synapses and/or to a decrease in neuronal inhibition^3,4^. Primary cultures of dissociated neurons and/or organotypic hippocampal slice cultures are good in vitro models to study the response to epileptogenic stimulation^3^. These model systems, coupled to the use of electrophysiology techniques, allow investigating the mechanism of action of anti-epileptic drugs or molecules that act at the receptor and channel levels^3^. One of the most common stimuli used to induce in vitro a SE-like condition is the incubation of cultured neurons or tissue slices in a Mg^2+^-free solution, which allows the activation of NMDA receptors due to the removal of the Mg^2+^ blockade of the receptor channels^3,5–12^. Studies performed in hippocampal slices incubated in a Mg^2+^-free solution showed synchronized neuronal activity, as determined with extracellular field potential recordings^3,12^, which is tightly related to alterations in synaptic transmission^13^. Importantly, the spontaneous epileptiform activity recorded in the latter model becomes resistant to benzodiazepines after prolonged periods^3^. Therefore, neuronal cell cultures and hippocampal slices (both acute preparations and organotypic cultures) are considered valuable tools to study the cellular and molecular alterations in synaptic connectivity and in plasticity mechanisms associated with diseases of the brain, including epilepsy.

Most studies using neuronal cultures as a model to study epileptogenesis are mainly focused on the analysis of the response during incubation in a Mg^2+^-free salt solution, which enhances neuronal activity and may resemble the *Status Epilepticus* (SE) period (e.g.^6,14,15^). In the present work we analyzed the alterations in the physiology of cultured hippocampal neurons incubated transiently in the absence of Mg^2+^ ([Mg^2+^]_0_), and then returned to a [Mg^2+^]-containing salt solution to model the period after SE. The data obtained by single cell calcium imaging analysis and whole-cell current clamp recordings showed that after induction of epileptogenesis, when neurons were returned to a solution containing magnesium, they were prone to develop spontaneous, recurrent and synchronous activity. This type of activity is comparable with the neuronal activity observed in the chronic phase of epilepsy, both in animal models of the disease and in human patients. Overall, the results of this study show that transient incubation of cultured hippocampal neurons in [Mg^2+^]_0_ can be used as a model of epileptogenesis. This model will be useful to investigate the synaptic mechanisms associated with epileptogenesis and to test new drugs for the treatment of epilepsy in different stages of the disease.

## Results

### Incubation of hippocampal neurons in [Mg^2+^]_0_ medium induces epileptiform activity

**Figure 1:**
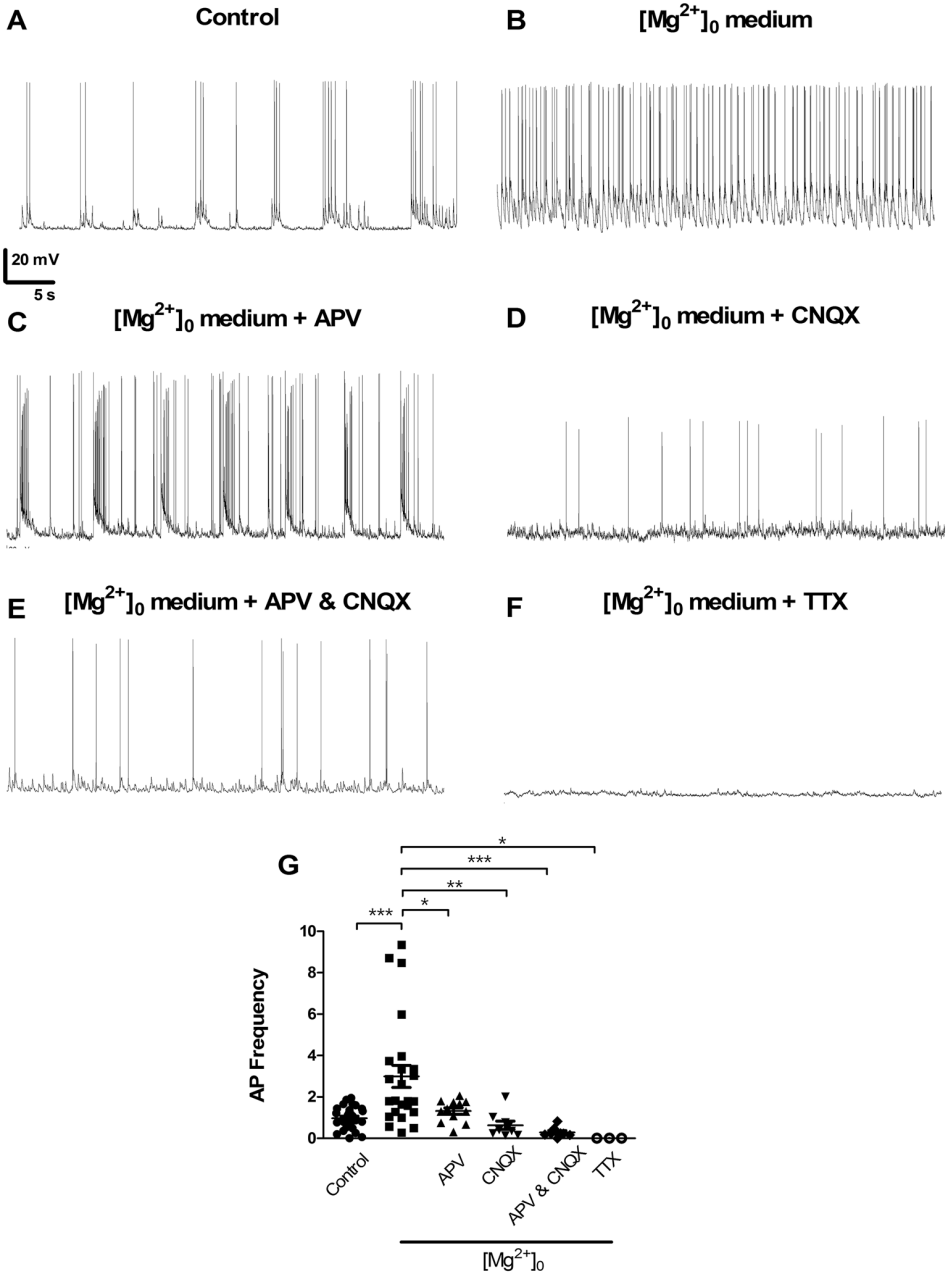
The increased neuronal firing upon treatment in [Mg^2+^]_0_ medium is glutamate receptor-dependent. The cells were incubated for 15 min in Mg^2+^-free solution in the absence of drugs, and recordings were performed in the same buffer for 25 min under the conditions indicated below. Representative recordings in control conditions (n = 24) and in [Mg^2+^]_0_ medium (n = 20) are shown in panels (**A**, **B**), respectively. Representative recordings performed in [Mg^2+^]_0_ medium and in the presence of APV (50 µM; NMDAR inhibitor) (n = 10), CNQX (20 µM; AMPAR inhibitor) (n = 9) and CNQX (20 µM) and APV (50 µM) (n = 8) are shown in panels (**C**–**E**), respectively. (**F**) Representative recording in SE condition in the presence of TTX (500 nM) (n = 3). (**G**) Summary data of action potential firing frequency under the different experimental conditions. The results were normalized for the mean frequency of action potential detected under control conditions. The results represent the fold change (mean ± SEM) of at least 3 independent experiments performed in distinct preparations. *p < 0.05, **p < 0.01, ***p < 0.001; one-way ANOVA followed by Bonferroni test.

To validate the transient incubation in [Mg^2+^]_0_ medium as an experimental strategy to model *Status Epilepticus* in vitro we analyzed the frequency of action potential firing in high density primary cultures of rat hippocampal neurons, using whole-cell current clamp electrophysiology. Neuronal activity was also measured in hippocampal neurons incubated in control salt solution. An approximately threefold increase in the frequency of action potentials was observed in neurons incubated in [Mg^2+^]_0_ medium (Fig. 1A,B,G), confirming that this experimental condition induces the development of epileptiform discharges.

In additional experiments we characterized the role of AMPA (AMPAR) and NMDA (NMDAR) receptors in the firing of action potentials in hippocampal neurons incubated in the absence of Mg^2+^. Hippocampal neurons were incubated in [Mg^2+^]_0_ medium for 15 min and afterwards whole-cell current clamp recordings were performed in the same medium and in the presence or absence of the following inhibitors: APV (50 μM; NMDAR antagonist) and/or CNQX (20 μM; AMPAR antagonist), and TTX (500 nM; blocker of voltage-gated Na^+^ channels). The increase in the frequency of firing of action potentials in [Mg^2+^]_0_ medium when compared with the control (Fig. 1A,B,G) was abolished upon incubation with APV, CNQX or with the two drugs together (Fig. 1C–E,G). As expected, TTX completely blocked the firing activity recorded in cells maintained in the absence of Mg^2+^ (Fig. 1F,G).

### Transient incubation in epileptogenic conditions induces synchronous neuronal activity and the development of spontaneous [Ca^2+^]_i_ oscillations

An episode of continuous seizure activity is sufficient to induce temporal lobe epilepsy (TLE) in diverse mammalian species^16^, and accordingly the occurrence of de novo SE is thought to contribute to development of TLE in humans^17^. To investigate how the bursts of activity induced by transient incubation of hippocampal neurons in a medium lacking Mg^2+^ affect the activity of the cells, we performed single-cell [Ca^2+^]_i_ imaging with the Fluo-4 fluorescent calcium indicator. After incubation of hippocampal neurons in [Mg^2+^]_0_ medium for 30 min the cells were further incubated in control salt solution containing Mg^2+^. Bursts of neuronal activity are expected to increase the [Ca^2+^]_i_ and this approach allows assessing the behaviour of all cells present in a given field of the microscope.

**Figure 2:**
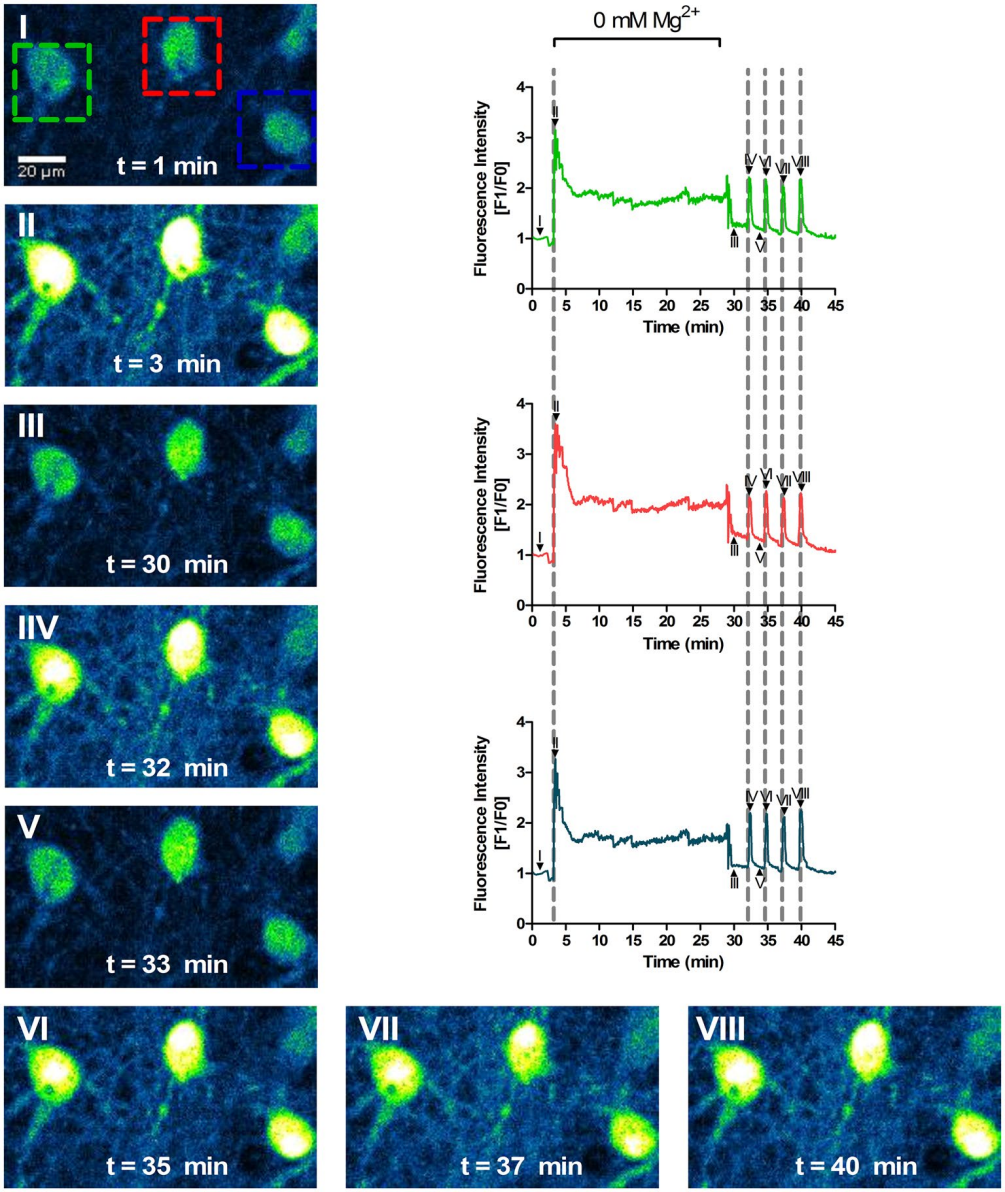
[Mg^2+^]_0_ increases the [Ca^2+^]_i_ and induces the development of spontaneous calcium oscillations. Cultured hippocampal neurons (15 DIV) were analysed by Single-Cell Calcium Imaging using the fluorescent Ca^2+^ indicator Fluo-4, by Spinning Disk microscopy. The cells were initially incubated under control conditions (with 2 mM Mg^2+^) for 3 min to determine the baseline levels of calcium. After 3 min, the medium was replaced with [Mg^2+^]_0_ medium for the indicated stimulation period. At 30 min, the medium was replaced by control salt solution for an additional period of 15 min. Fluo-4 fluorescence was recorded for the duration of the incubation. For each time point the results are represented as the normalized intensity of Fluo-4 fluorescence (Fluorescence for a given time point divided by the baseline fluorescence). The time courses represent the [Ca^2+^]_i_ changes in the neurons indicated by a coloured square. The images (I–VIII) represent key time points of the Fluo-4 fluorescence recording and their position in the traces is also shown. The dashed lines portray the synchronous neuronal calcium fluctuation pattern. A video of a representative experiment is shown in Supplementary Video S1.

Representative [Ca^2+^]_i_ profiles for three different neurons in the same field after transient incubation in [Mg^2+^]_0_ medium are presented in Fig. 2. The results show a rapid increase in the [Ca^2+^]_i_ immediately after incubation of hippocampal neurons in [Mg^2+^]_0_ medium. This [Ca^2+^]_i_ response was transient, and the initial peak was followed by a decrease towards a plateau which was maintained until the end of the incubation period in the epileptogenic stimulus (t = 30 min). Replacement of the [Mg^2+^]_0_ medium with the control salt solution induced an additional reduction in the [Ca^2+^]_i_, down to levels similar or slightly above those observed under resting conditions. However, as the [Ca^2+^]_i_ profile of the 3 neurons in Fig. 2 shows, the cells developed spontaneous and synchronous calcium transients for the rest of the experiment. This synchronous [Ca^2+^]_i_ response cannot be attributed to a toxic effect of the transient incubation in [Mg^2+^]_0_ medium since no significant cell death was observed when analysed 8 h later (Figure S1). In contrast, longer incubations in [Mg^2+^]_0_ medium induced cell death when determined 8 h later by analysis of nuclear morphology.

### Characterization of the [Ca^2+^]_i_ transients induced by 30 minutes of incubation under epileptogenic conditions

**Figure 3:**
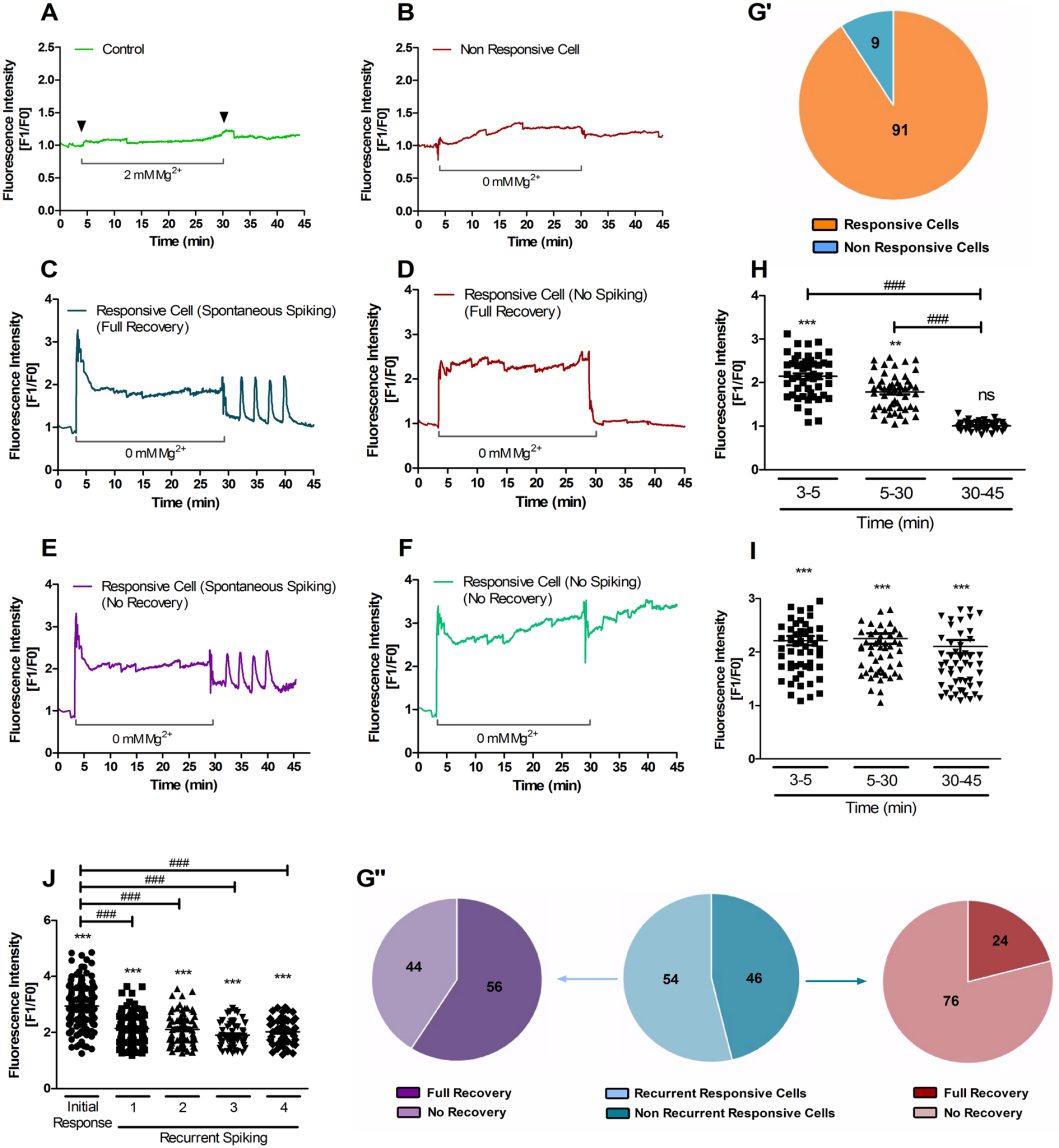
Characterization of [Ca^2+^]_i_ transients induced by incubation of hippocampal neurons in [Mg^2+^]_0_ medium for 30 min. (**A**–**F**) Cultured hippocampal neurons (15 DIV) were analysed by single cell calcium imaging using the Fluo-4 fluorescent Ca^2+^ indicator, with Spinning Disk microscopy. The cells were initially incubated in control salt solution containing 2 mM Mg^2+^ for 3 min to determine the baseline level of fluorescence. After 3 min, the solution was replaced with [Mg^2+^]_0_ medium for the indicated period. In the experiments described in panel (**A**), where indicated by the arrows the Mg^2+^-containing buffer was replaced by a solution with the same composition. At t = 30 min, the buffer was replaced by control salt solution for an additional period of 15 min. Fluo-4 fluorescence was recorded for the duration of the experiment. For each time point the results are represented as the normalized intensity of Fluo-4 fluorescence (Fluorescence for a given time point divided by the baseline fluorescence). The analyses represent the different types of response to the incubation in [Mg^2+^]_0_ medium. (**G**′, **G″)** Pie chart representation of the diversity of responses to incubation in [Mg^2+^]_0_ medium as a percentage of the total number of cells with the indicated pattern of response. (**H**, **I)** A comparative analysis of the [Ca^2+^]_i_ response profile was made between the cells that showed a recovery to basal calcium levels (**C** and **D; H)** and the cells that showed a partial recovery [e.g. **E** **]** or no recovery [e.g. (**F)**]**.** In the latter case the results are plotted together in panel (**I)**. (**J)** Calcium oscillations were comparatively analysed to determine the differences between the initial response to the incubation in [Mg^2+^]_0_ medium and the spontaneous transients that developed after the stimulus (in control salt solution). Results are the mean ± SEM of at least three independent experiments performed in distinct preparations. ***p < 0.001, ^###^*p* < 0.001; Repeated measures ANOVA followed by Dunnett’s (*) and/or Bonferroni (^#^) test. ns, not significantly different.

A detailed analysis of the [Ca^2+^]_i_ responses to transient exposure of hippocampal neurons to epileptogenic conditions, showed some heterogeneity in the behaviour of the cells. Figure 3 (panels A-F) summarizes the diversity of responses detected after incubation of hippocampal neurons in [Mg^2+^]_0_ medium for 30 min (SE-like period) followed by incubation in control salt solution. Among the cells that were analysed (n = 153 cells), 91% displayed an increase in the [Ca^2+^]_i_ when incubated in [Mg^2+^]_0_ medium (Fig. 3G′) and 54% of these neurons developed spontaneous calcium oscillations upon further incubation in control salt solution; the remaining population of cells did not develop this phenotype. The amplitude of the spontaneous calcium transients was 20% lower than the initial response to incubation in [Mg^2+^]_0_ medium (Fig. 3J) and remained constant throughout the experiment.

In the group of cells that developed spontaneous [Ca^2+^]_i_ oscillations following transient incubation in [Mg^2+^]_0_ medium, 56% of the neurons recovered the basal [Ca^2+^]_i_ during the period between each event (Fig. 3C). A lower percentage (24%) of the cells that did not develop spontaneous [Ca^2+^]_i_ transients showed a recovery of the calcium levels (Fig. 3G″). The cells that recovered the [Ca^2+^]_i_ after transient incubation in [Mg^2+^]_0_ medium (Fig. 3C,D) showed an initial increase in Fluo-4 fluorescence of ~128% (Fig. 3H). This initial response was followed by a slow decrease towards a plateau at ~80% above the resting Fluo-4 fluorescence (at 5–30 min; Fig. 3H), and the [Ca^2+^]_i_ further decreased upon incubation of the cells in control salt solution (Fig. 3H). In comparison, the cells that did not recover from the incubation period in [Mg^2+^]_0_ medium (Fig. 3E and F) showed a similar (~114%) but sustained increase in Fluo-4 fluorescence when incubated under conditions that model SE (Fig. 3I), and the effects were maintained even after incubation with control salt solution (Fig. 3I). A subpopulation of cells in the latter group displayed [Ca^2+^]_i_ oscillations when incubated in the control salt solution after exposure to the [Mg^2+^]_0_ medium (Fig. 3E). Together, these results indicate that the magnitude of the initial [Ca^2+^]_i_ increase following incubation in [Mg^2+^]_0_ medium does not account for the impairment in [Ca^2+^]_i_ homeostasis mechanisms. However, the delayed [Ca^2+^]_i_ response to the incubation under the latter conditions (after the initial rapid increase) correlated well with the pattern of response when the epileptogenic stimulus was removed.

### Characterization of the [Ca^2+^]_i_ transients induced by 15 minutes of incubation under epileptogenic conditions

**Figure 4:**
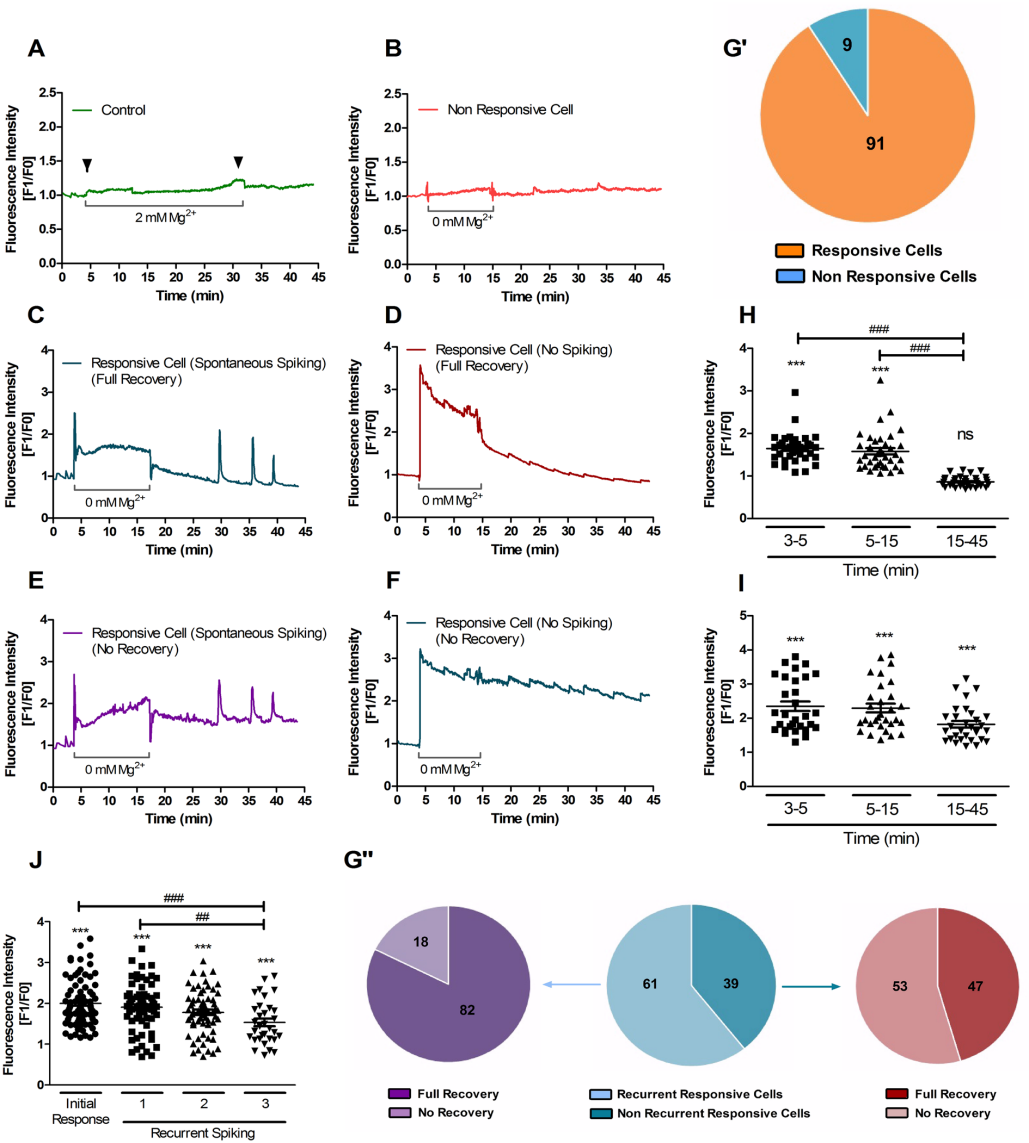
Characterization of [Ca^2+^]_i_ transients induced by incubation of hippocampal neurons in [Mg^2+^]_0_ medium for 15 min. (**A–F)** Cultured hippocampal neurons (15 DIV) were analysed by single cell calcium imaging using the fluorescent Ca^2+^ indicator Fluo-4, with Spinning Disk microscopy. The cells were initially incubated in control salt solution containing 2 mM Mg^2+^ for 3 min to determine the baseline fluorescence level. After 3 min, the solution was replaced with [Mg^2+^]_0_ medium for the indicated period of time. At t = 15 min the buffer was replaced by control salt solution for an additional period of 30 min. In the experiment described in panel **A**, where indicated by the arrows the Mg^2+^-containing salt solution was changed to a buffer with the same composition. Fluo-4 fluorescence was recorded for the duration of the experiment. For each time point the results are represented as the normalized intensity of Fluo-4 fluorescence (fluorescence for a given time point divided by the baseline fluorescence). The analyses represent the different types of response to the [Mg^2+^]_0_ medium. (**G′-G″)** Pie chart representation of the types of response to [Mg^2+^]_0_ medium in percentage of total cells with the indicated pattern of response. (**H, I**) A comparative analysis of the [Ca^2+^]_i_ response profile was made between cells that were able to recover to basal calcium levels, (**C** and **D**;** H**) and the cells that did not (**E** and **F**;** I**). (**J**) [Ca^2+^]_i_ transients were comparatively analysed to determine the differences between the initial response to the incubation in [Mg^2+^]_0_ medium and the spontaneous oscillations that developed after the stimulus (in Sham medium). Results are the mean ± SEM of at least three independent experiments performed in distinct preparations, ***p < 0.001, ^##^*p* < 0.01, ^###^*p* < 0.001; Repeated measures ANOVA followed by Dunnett’s (*) and/or Bonferroni test (^#^). ns, not significantly different.

In order to determine whether different incubation periods in [Mg^2+^]_0_ medium could translate into distinct epileptogenic phenotypes, with an impact on the spontaneous calcium transient profiles, we recorded the [Ca^2+^]_i_ changes in cultured hippocampal neurons in control salt solution after a 15 min pre-incubation in the absence of [Mg^2+^]_0_. Single-cell calcium imaging using Fluo-4 and Spinning Disk microscopy showed an increase in the [Ca^2+^]_i_ when the cells were incubated in [Mg^2+^]_0_ medium, similar to the results in Figs. 2 and 3. After 15 min of incubation under these conditions the cells were exposed to control salt solution for 30 min, and the results showed different [Ca^2+^]_i_ response profiles (Fig. 4A–F). Among the cells analysed (n = 129 cells), 91% responded to the initial incubation in [Mg^2+^]_0_ medium with an increase in the [Ca^2+^]_i_ (Fig. 4G′), in agreement with the results of Fig. 3. Among these, 61% developed spontaneous calcium oscillations when returned to control salt solution (at t = 15 min). The spontaneous amplitude of the [Ca^2+^]_i_ transients decreased by ~20% from the first to the third event, indicating a progressive loss of intensity of the spontaneous [Ca^2+^]_i_ oscillations with time (Fig. 4J).

When spontaneous [Ca^2+^]_i_ oscillations were observed, 82% of the cells showed a recovery of the basal [Ca^2+^]_i_ levels between events. In contrast, in the population of hippocampal neurons that did not develop spontaneous calcium transients, only 47% of the cells recovered the basal calcium levels (Fig. 4G″). To determine whether the [Ca^2+^]_i_ dysregulation observed in the latter group of cells was correlated with an increased Ca^2+^ entry in response to the initial exposure to [Mg^2+^]_0_ medium, we compared the two sets of data. The cells that fully recovered the [Ca^2+^]_i_ in control salt solution after the 15 min in epileptogenic conditions (Fig. 4C,D) showed an initial increase in Fluo-4 fluorescence of ~64% when exposed to [Mg^2+^]_0_ medium (Fig. 4H), followed by a decrease (~10%) towards a plateau (Fig. 4H). In comparison, the cells in which the [Ca^2+^]_i_ did not recover in control medium (Fig. 4E,F) showed a similar (~58%) increase in Fluo-4 fluorescence after stimulation with the [Mg^2+^]_0_ medium (Fig. 4I), but in this case the [Ca^2+^]_i_ response was slightly increased during the rest of the stimulus (Fig. 4I). Under the latter conditions, the [Ca^2+^]_i_ levels remained unchanged for the rest of the experiment in control salt solution (Fig. 4I). A subpopulation of these cells displayed [Ca^2+^]_i_ oscillations when incubated in the control salt solution after transient exposure to [Mg^2+^]_0_ medium (Fig. 4E).These results show no correlation between the magnitude of the [Ca^2+^]_i_ response to the [Mg^2+^]_0_ buffer and the delayed dysregulation of the mechanisms that contribute to the maintenance of the resting [Ca^2+^]_i_. Similar to the results obtained for longer SE periods, it was the delayed [Ca^2+^]_i_ response to the incubation in [Mg^2+^]_0_ medium (after the initial rapid increase) that correlated with the pattern observed after the epileptogenic-like period.

**Figure 5:**
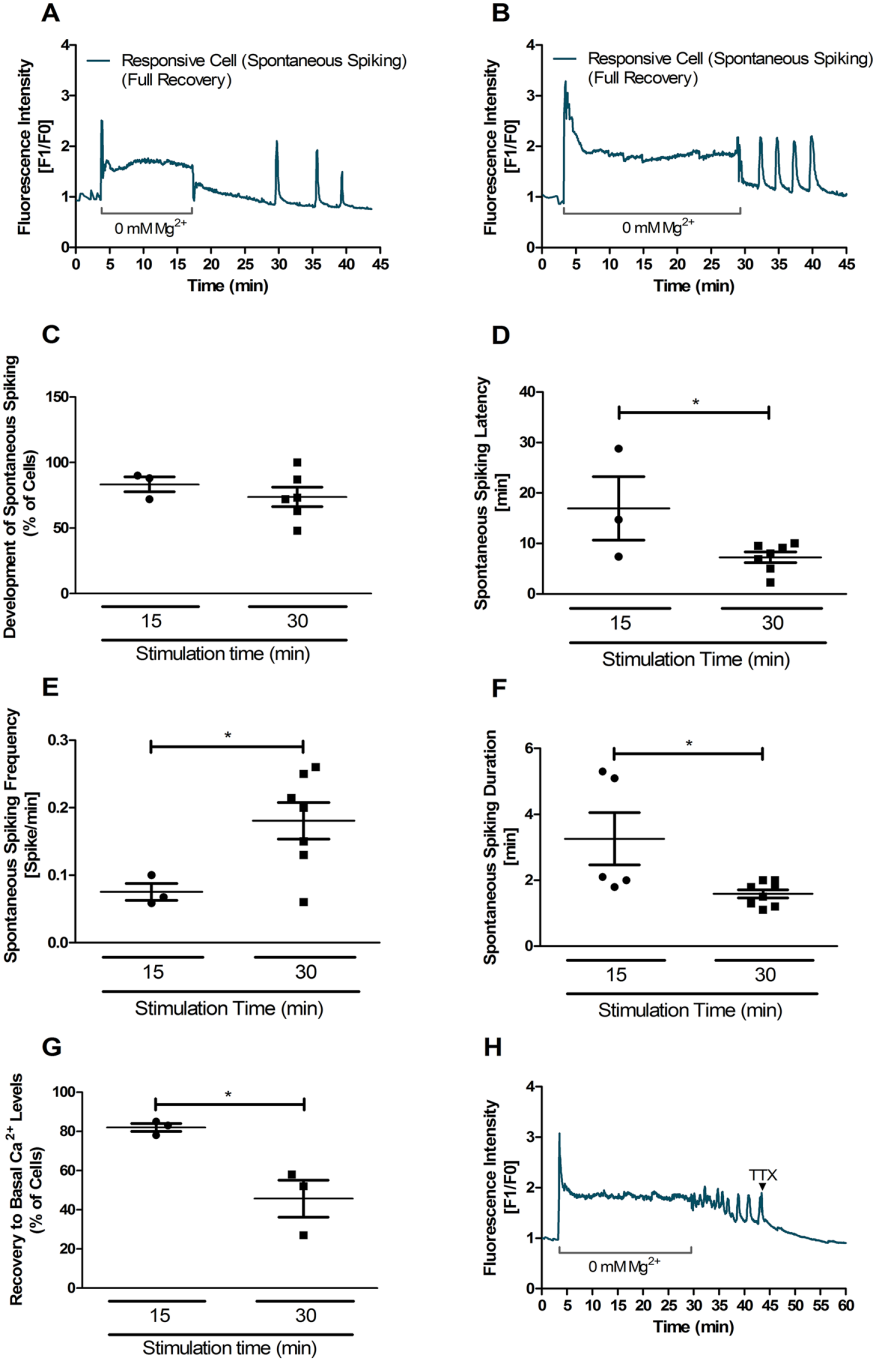
Different periods of incubation in [Mg^2+^]_0_ medium induce spontaneous calcium oscillations with distinct characteristics. (**A**,** B**) Cultured hippocampal neurons (15 DIV) were analysed by single cell calcium imaging using the fluorescent Ca^2+^ indicator Fluo-4, with Spinning Disk microscopy. The cells were initially incubated in control salt solution for 3 min to determine the baseline fluorescence level. The solution was then replaced with [Mg^2+^]_0_ medium and the cells were further incubated under these conditions for 15 min (**A**) or 30 min (**B**). After this incubation period the cells were further incubated in control salt solution. Fluo-4 fluorescence was recorded for the duration of the experiment and for each time point the results are presented as the normalized intensity of Fluo-4 fluorescence (Fluorescence for a given time point divided by the baseline fluorescence). (**C**,** G**) Analysis of the response profile of cells that developed spontaneous calcium transients after 15 min (n = 60 cells) and 30 min (n = 90 cells) of stimulation with [Mg^2+^]_0_ medium. The following parameters were analysed: (**C**) Percentage of cells that develop spontaneous calcium transients, (**D**) latency period, (**E**) frequency and (**F**) duration of calcium transients, and (**G**) percentage of cells that recover to basal calcium levels. (**H**) Similar experiment to that depicted in B but with TTX (1 µM) added at the indicated time point. The results are representative of at least three independent experiments performed in distinct preparations. Values are the mean ± SEM. Statistical analysis was performed by unpaired Student’s *t* test, *p < 0.05.

Although the two protocols used to induce epileptiform-like activity, for 15 min and 30 min, induced spontaneous [Ca^2+^]_i_ transients (Fig. 5A–C), there were clear differences in the results obtained. Thus, cells that underwent incubation for 30 min displayed a 56% shorter latency to develop spontaneous calcium oscillations when compared with cells that were incubated for only 15 min (Fig. 5D). The frequency of spontaneous calcium transients was 58% higher in cells that were subjected to 30 min of [Mg^2+^]_0_ medium (Fig. 5E) but the events displayed a shorter duration (39% difference) under these conditions (Fig. 5F). These results indicate that the longer period of incubation in the absence of Mg^2+^ induces more frequent but shorter-lasting spontaneous calcium transients. Interestingly, about 82% of the cells that were exposed to [Mg^2+^]_0_ medium for 15 min recovered the [Ca^2+^]_i_ to basal levels, while only about 46% of the cells showed the same type of behaviour upon a 30 min incubation (Fig. 5G). This suggests that the cells subject to the latter conditions were less efficient in maintaining the [Ca^2+^]_i_ homeostasis.

To further investigate the mechanisms underlying spontaneous [Ca^2+^]_i_ oscillations in hippocampal neurons subjected to transient incubation in [Mg^2+^]_0_ medium for 30 min, the cells were further incubated in control salt solution, to allow the development of calcium transients, before perfusion with tetrodotoxin (TTX), a blocker of voltage-gated Na^+^ channels. Figure 5H shows that addition of TTX (1 µM) to the medium after the third spontaneous calcium transient completely abolished [Ca^2+^]_i_ oscillations, indicating that this phenomenon is dependent on neuronal activity.

### Characterization of rhythmic electrical activity in hippocampal neurons transiently incubated in the absence of Mg^2+^

The spontaneous and synchronized [Ca^2+^]_i_ transients, sensitive to TTX, recorded in cultured hippocampal neurons incubated in [Mg^2+^]_0_ medium and later perfused with a control salt solution (Fig. 2, 3, 4 and 5), suggest that transient exposure of the cells to conditions that elicit SE-like activity in vitro enhances neuronal excitability. This was further investigated using whole-cell patch clamp.

**Figure 6:**
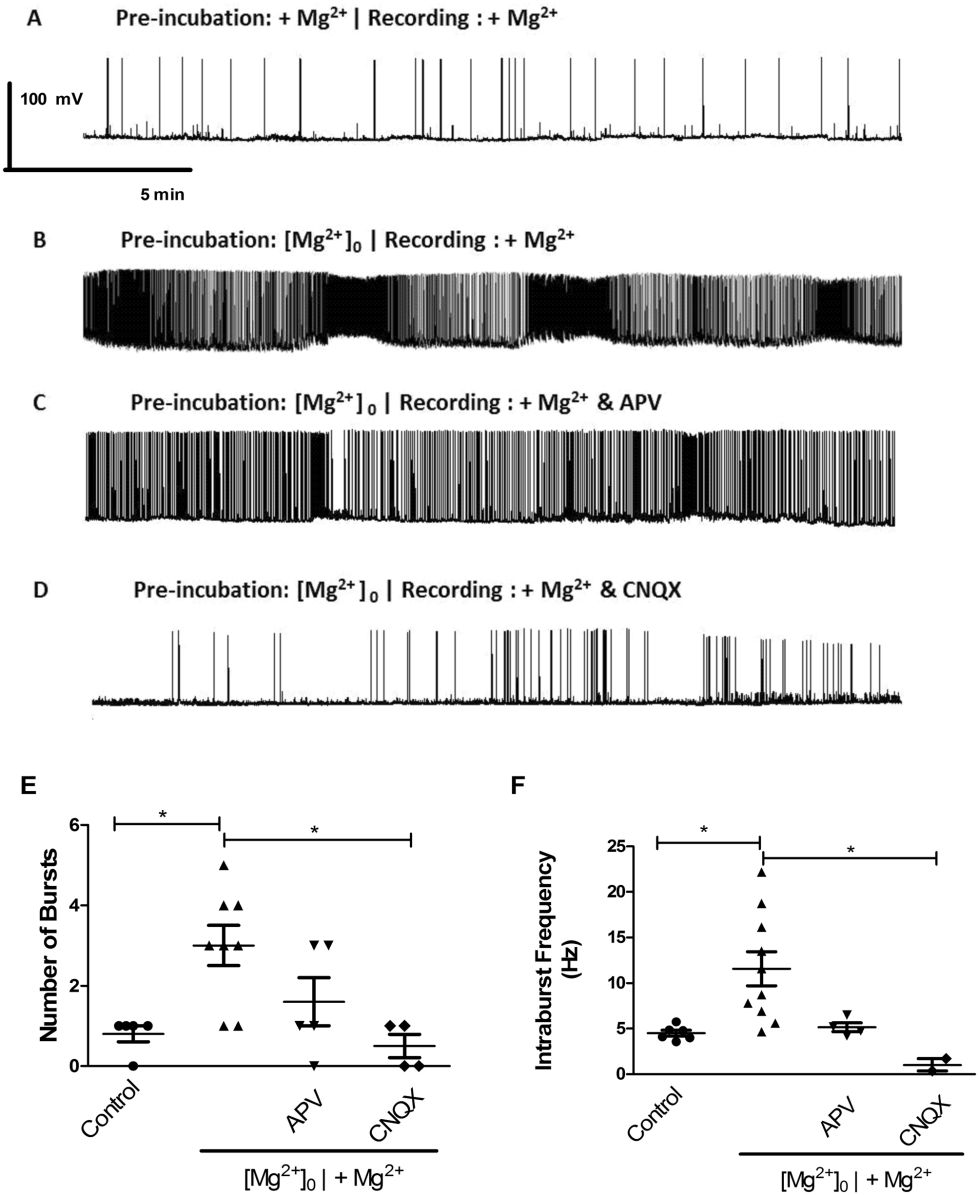
The rhythmic bursting activity induced by incubation in [Mg^2+^]_0_ medium depends on the activity of ionotropic glutamate receptors. (**A–D**) Representative 25 min recordings after incubation in control salt solution (**A**), or after 15 min of stimulation in [Mg^2+^]_0_ medium mimicking SE (**B–D**). Recordings were performed in control salt solution, in the absence (**B**) or in the presence of APV (50 µM) (**C**) or CNQX (20 µM) (**D**). The average number of bursts during the 25 min recording is plotted in panel (**E**). Panel (**F**) shows the mean frequency of intraburst events. Burst analysis was performed using the Poisson Surprise method. The results are the mean ± SEM of 10 cells from at least 3 independent preparations. Statistical analysis was performed by one-way ANOVA followed by the Bonferroni test (*p < 0.05).

Hippocampal neurons were incubated for 30 min in [Mg^2+^]_0_ medium and post-incubated in control salt solution while recorded in whole-cell current clamp. As depicted in Fig. 6B, transient incubation in a [Mg^2+^]_0_ medium induced a massive increase in the firing frequency of the neurons and a pattern of rhythmic bursting activity characterized by an even higher firing frequency and clear sustained depolarization. In a 25 min recording, there was an average of 3 bursts occurring. During the bursting period, the frequency of action potentials was 11.6 Hz (Fig. 6F) while in the whole recording a frequency of 0.6 Hz was calculated (not shown). These firing bursts show a similar time course (corresponding to 3.3 MHz) when compared with the synchronized calcium oscillations observed after transient incubation of hippocampal neurons in a [Mg^2+^]_0_ medium (see Fig. 2, 3, 4 and 5) and further support the hypothesis that this is a good in vitro model of *Status Epilepticus*. However, in the electrophysiology experiments the number of cells analyzed was much lower when compared with the Fluo-4 measurements, preventing a more detailed analysis of the different patterns of cellular behavior.

Control experiments showed that the pattern of activity recorded in hippocampal neurons maintained always in control salt solution (with 2 mM Mg^2+^) was stable and low throughout the recording period (Fig. 6A,E). Burst analysis using the Poisson Surprise method^18^ showed that during the 25 min of incubation in control salt solution after transient perfusion with [Mg^2+^]_0_ medium there was an increase of rhythmic bursts when compared with the control condition (Fig. 6A,B,F).

In additional experiments, we performed a pharmacological characterization of the bursts of action potentials in hippocampal neurons subjected to a transient incubation in the absence of Mg^2+^, followed by incubation in a solution containing a physiological [Mg^2+^]. The inhibitors of ionotropic glutamate receptors, APV and CNQX, decreased bursting activity, but the effect was statistically significant only in the experiments performed in the presence of the non-NMDA receptor antagonist (Fig. 6C,D). Both the average number of bursts (Fig. 6E) and the intraburst frequency of events (Fig. 6F) were significantly reduced by CNQX.

## Discussion

Neuronal cultures and brain slices are essential tools in the study of the cellular and molecular mechanisms underlying epileptogenesis since they allow studying alterations in synaptic connectivity in response to conditions of enhanced excitatory activity. Furthermore, they allow testing the effects of anti-epileptic drugs. One of the most common stimuli used to induce in vitro SE-like activity is the incubation of cultured neurons or brain slices in a Mg^2+^-free solution which enhances the activity of neuronal networks^3,5–12^. In this work we found that transient incubation of cultured hippocampal neurons under these conditions induces spontaneous and synchronized bursts of neuronal activity coupled to oscillations of the [Ca^2+^]_i_. Since these bursts of activity take place after the trigger has been removed, they may be considered an in vitro mimetic of seizure activity following SE although with a distinct time scale. The pattern of [Ca^2+^]_i_ and electrophysiology responses (including spiking activity) observed in cultured hippocampal neurons subjected transiently to conditions that generate epileptiform-like activity, further validates the [Mg^2+^]_0_ model as an experimental strategy to study epileptogenesis in vitro. Furthermore, the periods of SE-like activity tested are appropriate not only to study the cellular and molecular alterations during this stage of the disease, but also to investigate long-term changes induced by SE and the development of chronic epilepsy.

Incubation of cultured hippocampal neurons in [Mg^2+^]_0_ medium enhanced the frequency of action potentials and increased rapidly the [Ca^2+^]_i_ as determined with the fluorescent indicator Fluo-4. The increased bursting activity under the same conditions was previously reported in hippocampal and cortical slices^9,10,12^, hippocampal organotypic cultures^11^ and in hippocampal neuronal cultures^6,8^. In addition, we found that the network bursting observed in Mg^2+^-free solution was decreased in the presence of inhibitors of NMDA and non-NMDA (possibly AMPA) receptors, in accordance with previous observations^6^. The latter study also showed an increase in the probability of neurotransmitter release from nerve endings under the same conditions. The role of NMDA receptors in the increased excitability is likely to result from the effect of Mg^2+^ as a blocker of the receptor channel from the outside of the cell at a resting membrane potential^3,19^. AMPA and NMDA receptor inhibitors also prevent the increased neuronal activity during *Status Epilepticus*^6,20^, similar to the results obtained in this work.

Importantly, after incubation of hippocampal neurons under conditions that model SE in vitro, around ~50% of the cells developed spontaneous [Ca^2+^]_i_ transients. This spontaneous activity occurred during a period when the cells were no longer incubated in [Mg^2+^]_0_ medium, under conditions that mimic the physiological extracellular [Mg^2+^]. When hippocampal neurons were maintained in control salt solution throughout the entire experiment, no spontaneous oscillations were observed, indicating that this type of behaviour was not due to an artefact of the experimental settings, being instead evoked by the epileptogenic conditions. In addition, the spontaneous [Ca^2+^]_i_ oscillations were synchronous between the cells exposed to the SE stimulus and was blocked by TTX, an inhibitor of voltage-gated sodium channels, indicating that this process is dependent of neuronal activity. Accordingly, bursts of action potentials were also recorded in cultured hippocampal neurons in physiological [Mg^2+^] after a period of incubation in the absence of the cation, with a frequency similar to the [Ca^2+^]_i_ oscillations. The increased electrical activity recorded in hippocampal neurons after a period that model SE in vitro was particularly sensitive to inhibition by the non-NMDA receptor antagonist CNQX, which affected the number of bursts and the intraburst activity. Since the pattern of neuronal activity after transient incubation in the absence of Mg^2+^ was also sensitive to APV, an inhibitor of NMDA receptors, the effect of CNQX may be partly due to a decrease in AMPA receptor-mediated depolarization of the membrane which is required for activation of NMDA receptors^19^.

It is important to note that the frequency of action potentials decreased when hippocampal neurons were incubated in control salt solution after a period of exposure to conditions that model SE in vitro. This difference is probably due to the fact that after incubation in [Mg^2+^]_0_ the bursting activity alternates with interburst periods characterized by a lower frequency of AP firing. In the present work we also found (i) a significant reduction in bursting activity in the presence of CNQX or APV, and (ii) a greater decrease in frequency of APs in the presence of CNQX. These results contrast with those obtained in a previously reported study using hippocampal neurons exposed to [Mg^2+^]_0_ for 3 h and further incubated in culture medium supplemented with horse serum for 2 days. Under the latter conditions there was a reduction in spiking activity to approximately control levels in the presence of 25 μM APV, with no burst activity. Furthermore, although burst activity was observed in the presence of 10 μM CNQX it showed a shorter duration^20^. The latter response may arise from neuronal damage, as suggested by our results showing apoptotic death of hippocampal neurons after incubation in the absence of Mg^2+^ for periods longer than 30 min. However, if this is not the case, the differences between the two sets of data may indicate that the mechanisms underlying the epileptiform discharges change along the time of incubation in Mg^2+^-containing medium. These changes may result from adaptative processes present in neurons that allow adjusting their excitability depending on the activity of neuronal networks^21,22^. Such homeostatic mechanisms may account for responses recorded 2 days after transient incubation of neurons in [Mg^2+^]_0_ medium, the experimental conditions used in^20^, but not immediately after stimulation under conditions that induce SE-like activity (present work). Furthermore, it would be of interest to determine whether similar results are obtained in experiments using lower extracellular concentrations of Ca^2+^, within the more physiological range of 1.3–1.8 mM (2 mM CaCl_2_ was used in the experiments reported in this work)^23^, since Ca^2+^ has an effect on intrinsic neuronal excitability^24^.

The epileptic discharges after transient incubation of hippocampal neurons in [Mg^2+^]_0_ medium correlated with synchronized [Ca^2+^]_i_ oscillations. We also observed that different times of incubation under the latter conditions induced distinct [Ca^2+^]_i_ profiles in what concerns spontaneous oscillations. This shows a time-dependent effect of SE-like activity in the regulation of the [Ca^2+^]_i_ homeostasis.

Not all hippocampal neurons that showed an increase in the [Ca^2+^]_i_ following transient incubation in [Mg^2+^]_0_ developed calcium oscillations at a later point, when incubated in control salt solution. Although it is not clear why these cells showed a distinct behaviour, several hypothesis can be raised: (i) silent cells may be different since this culture is known to contain distinct types of neurons (e.g. glutamatergic and GABAergic neurons)^25,26^; (ii) cells that did not develop [Ca^2+^]_i_ oscillations may lack the minimum number of synaptic contacts required for synchronization of the calcium responses; (iii) the expression of different Ca^2+^ buffering mechanisms and/or surface receptors for glutamate may account for responses to transient incubation in [Mg^2+^]_0_. It is important to point out that the density of the neuronal culture is a crucial factor for this model. Indeed, a previous study showed that, despite manifesting increased EPSC frequency, cells from low-density cultures did not exhibit action potential bursting when maintained in zero magnesium conditions^6^.

Several studies have shown that the epileptogenic period is characterized by an alteration of [Ca^2+^]_i_ homeostatic mechanisms (reviewed in^27^), but the intracellular calcium dynamics in SE is still poorly characterized. The increase in the [Ca^2+^]_i_ in SE may be coupled to activation of genes coding for growth factors, such as brain-derived neurotrophic factor (BDNF) and fibroblast growth factor (FGF), and to changes in the expression of cytoskeletal proteins and in glutamate receptors^28,29^. Together, these alterations are thought to contribute to the development of epileptic circuits^30^. In accordance with their key role in epilepsy, voltage-dependent Ca^2+^ channels, together with Ca^2+^-binding proteins, have been reported to be involved in all stages of the pathogenesis of epilepsy^31,32^. The key role of Ca^2+^ in epileptogenesis suggests that the [Ca^2+^]_i_ rise, with a consequent activation of downstream signalling mechanisms, may be important in the induction of the bursts of synaptic activity and [Ca^2+^]_i_ oscillations after transient incubation of hippocampal neurons in [Mg^2+^]_o_ medium. This effect was more remarkable when longer periods in the absence of Mg^2+^ were tested, and may explain the shorter lag-phase for initiation of the [Ca^2+^]_i_ transients upon removal of the buffer that induces SE-like activity, as well as their increased frequency. The distinct profiles of the spontaneous [Ca^2+^]_i_ oscillations depending on the duration of incubation in the absence of Mg^2+^ may be due to differential activation of Ca^2+^-dependent signalling mechanisms during this period. The distinct duration of sustained Ca^2+^ signals during the period of incubation in [Mg^2+^]_o_ medium may be important in determining which transcription factors are preferentially activated^33,34^. Also, the Ca^2+^- and calmodulin-dependent protein kinase II can undergo inactivation by autophosphorylation on threonine 305/306 after prolonged activity, with an impact on downstream mechanisms such as gene expression^35–37^.

The Fluo-4 calcium imaging in single cells also showed that the spontaneous [Ca^2+^]_i_ oscillations recorded after incubation of hippocampal neurons in [Mg^2+^]_o_ were synchronized. This synchronized activity may be explained based on the numerous synaptic connections established by hippocampal neurons in primary cultures (e.g.^38^) and resemble the coordinated hyperactivity of a population of glutamatergic neurons that underlie seizures^39–41^. The excessive activity of neuronal networks may also arise from a deficient neuronal inhibition due to an insufficient GABA_A_ receptor mediated neurotransmission^42,43,47^.

The results discussed above showed that incubation of hippocampal neurons under conditions that model SE induces a massive influx of Ca^2+^ downstream of NMDA receptor activation. As expected, the longer the cells were exposed to SE the higher was cell death due to excitotoxic mechanisms resulting from an [Ca^2+^]_i_ overload. Excitotoxicity is characterized as a deleterious effect resulting from an excessive or prolonged activation of glutamate receptors by excitatory signals. This induces a multitude of deleterious effects, such as impairment of intracellular calcium homeostasis, compromised organelle functions, increase in nitric oxide and free radical production, persistent activation of proteases and kinases, increase in expression of pro-death transcription factors and immediate early genes^44^. In particular, NMDA receptors play a fundamental role in the development of excitoxicity due to their high Ca^2+^ permeability^45,48^.

In summary, the work presented here shows that transient incubation of hippocampal neurons in [Mg^2+^]_0_ medium leads to the development of spontaneous bursts of activity after returning them to a salt solution containing Mg^2+^. This can be observed by recording action potentials in single cells with whole-cell current clamp electrophysiology and by measuring the [Ca^2+^]_i_ in populations of neurons. Whether similar responses can be induced by transient depolarization of cultured neurons with KCl, or other stimuli, remains to be investigated. These can be excellent tools to elucidate the molecular mechanisms underlying epileptogenesis at an early stage of the disease and to test new drugs for epilepsy.

## Methods

### Cultures of hippocampal neurons

Cultures of hippocampal neurons with a density of 8.0 × 10^4^ cells/cm^2^ were prepared from Wistar rat embryos (E18-E19)^46^. Animals were obtained from the CNC animal facility, and the project was approved by the institutional Animal Ethics Committee (ORBEA) as well as by the the National authorities (Direcção-Geral de Alimentação e Veterinária) (References 0421/000/000/2013 and 0421/000/000/2020). Experiments were performed according to the European Union Directive 2010/63/UE and the legislation Portaria n. 113/2013, issued by the Portuguese Government for the protection of animals used for experimental and other scientific purposes. Dams were anesthetized with isoflurane followed by decapitation, and embryos were then surgically removed and sacrificed by decapitation. Hippocampi were dissected from diencephalic structures and washed with Mg^2+^- free Hank’s balanced salt solution (HBSS, composed of: 5.56 mM KCl, 0.44 mM KH_2_PO_4_, 137 mM NaCl, 4.16 mM NaHCO_3_, 0.34 mM HEPES and 0.001% phenol red). The tissue was then digested with trypsin (0.06% in HBSS) for 15 min at 37 °C with gentle shaking, followed by a washing step with HBSS supplemented with 10% fetal bovine serum (Thermo Fisher Scientific) to stop trypsin activity. Hippocampi were then transferred to Neurobasal Medium (Thermo Fisher Scientific) supplemented with SM1 (1:50 dilution; Stem Cell Technologies, # 05711), 25 µM glutamate, 0.5 mM glutamine and 50 µg/ml gentamicin, and further dissociated with a pipette. The suspension was filtered (70 µm filter) and the cells were counted and plated on poly-D-lysine coated glass coverslips. Cultures were maintained for 15–16 days at 37 °C and with an atmosphere of 5% CO_2_/ 95% air. After 3 days in culture the medium was supplemented with FDU (5-fluoro-2′-deoxyrudine, 10 µM; Sigma Aldrich) and at day 7 in culture fresh culture medium lacking glutamate (one third of the total volume) was added to the wells.

### Induction of epileptiform* activity*

Cultured hippocampal neurons (15 DIV) were incubated with a [Mg^2+^]_0_ medium (148 mM NaCl, 2.5 mM KCl, 2 mM CaCl_2_, 10 mM Glucose, 10 mM HEPES, pH 7.4) for 15 min to 2 h, as indicated in the figure captions. For experimental conditions with a post-incubation period, the [Mg^2+^]_0_ medium was replaced by the control salt solution (with same composition of the [Mg^2+^]_0_ medium and supplemented with 2 mM MgCl_2_) and analyses were performed during the indicated period. The same solution was used for control experiments in which the cells were not exposed to [Mg^2+^]_0_ medium. The [Mg^2+^]_0_ medium and the control salt solution (containing 2 mM MgCl_2_) had an osmolarity of approximately 300 mOsm.

### Nuclear morphology analysis

After stimulation neurons were fixed in 4% sucrose/ 4% paraformaldehyde in PBS. After fixation, the cells were washed twice with ice-cold PBS and incubated with the fluorescent dye Hoechst 33342 (1 µg/mL) for 10 min. After nuclear staining the coverslips were washed twice with ice-cold PBS and mounted on glass slides with fluorescence mounting medium (DAKO). Images were acquired on an Axio Observer 2.1 fluorescence microscope coupled to an Axiocam HRm digital camera, using a 40×, 0,95 NA objective and the ZEN Blue software 2012. For each experimental condition three coverslips were analysed (at least 200 cells per coverslip were counted), and at least three independent experiments were performed, using distinct cell preparations.

### Single cell [Ca^2+^]_i_ imaging using Fluo-4 calcium indicator

Hippocampal neurons cultured on coverslips at a density of 8.0 × 10^4^ cells/cm^2^ were pre-incubated for 30 min with Fluo-4 AM (5 µM) (Thermo Fisher Scientific) prepared in control salt solution supplemented with 0.2% Pluronic F-127 (Thermo Fisher Scientific). The coverslips were then washed with control salt solution, mounted on a chamber filled with the same buffer and placed on the microscope stage, where they were kept at 37 °C. Fluo-4 baseline fluorescence was recorded for 3 min, after which the solution was replaced by [Mg^2+^]_0_ medium and the cells were further incubated for 15 or 30 min. Finally, the [Mg^2+^]_0_ medium was replaced by control salt solution for the rest of the experiment. When appropriate, control salt solution supplemented with 1 µM TTX (tetrodotoxin, 500 nM; Tocris) was added to the cells after the third spontaneous [Ca^2+^]_i_ oscillation. The preparation was excited at 488 nm using a solid-state 100mW laser. Fluo-4 fluorescence was recorded through a Zeiss Cell Observer Spinning Disk microscope with a Plan-Apochromat 20 × , 0.8 NA objective coupled to the highly sensitive Electron Multiplying-CCD Evolve Delta (Teledyne Photometrics) camera and a Definite Focus system that allows long-term time-lapse experiments without any focus drifts. Fluo-4 fluorescence was recorded in intervals of 10 s for the first 3 min (baseline). For the rest of the experiment fluorescence was recorded in intervals of 3 s. Analysis was performed using the ImageJ analysis software.

### Whole-cell current clamp electrophysiology

Hippocampal neurons (DIV 15) cultured on coverslips at a density of 8.0 × 10^4^ cells/cm^2^ were whole-cell current clamped at room temperature for 25 min in two sets of experiments. In the first set, cells were incubated in [Mg^2+^]_0_ medium (148 mM NaCl, 2.5 mM KCl, 2 mM CaCl_2_, 10 mM Glucose, 10 mM HEPES, pH 7.4) for 15 min and recorded in the same solution with or without the following inhibitors: TTX, APV (50 μM; Enzo Life Sciences) and CNQX (6-Cyano-7-nitroquinoxaline-2,3-dione, 20 μM; Tocris). In the second set, cells were incubated in [Mg^2+^]_0_ medium for 30 min and then post-incubated in control salt solution; recordings were performed during post-incubation in the presence or in absence of the inhibitors listed above. The pipette solution had the following composition (in mM): 100 K-gluconate, 20 KCl, 20 HEPES, 13.6 NaCl, 3 MgATP, 1 EGTA, 0.2 CaCl_2_ (pH 7.3, 300 mOsM). Recording electrodes (3–4 MOhm) were made from borosilicate glass capillaries pulled on a horizontal stage P-97 puller (Sutter Instruments). Seals (1–10 GΩ) were formed by applying gentle suction to pipettes. Recordings of spontaneous cell activity were performed using an Axon CNS Multiclamp 700B amplifier coupled to an Axon Digidata 1550 A acquisition board and pClamp software (version 10.6; Molecular Devices). Recordings were performed holding the current to maintain the cell potential at − 65 mV. The signals were low-pass filtered at 2.8 kHz and sampled at 25 kHz. Quantification was done through Clampfit (10.7; Molecular Devices).

### Statistical analysis

Statistical analysis was performed using GraphPad Prism 6. The results are presented as mean ± S.E.M. of at least three different experiments performed in independent neuron preparations. Statistical significance was calculated by one-way ANOVA or repeated measures ANOVA in the case of paired measurement, followed by Dunnett’s or Bonferroni’s post-test, or by Student’s *t* test, as indicated in the figures captions.

## Supplementary Information

Supplementary Information 1.

Supplementary Video 1.

## Data Availability

The authors have no restrictions on the availability of materials.
