# Supplementary material for: Transient incubation of cultured hippocampal neurons in the absence of magnesium induces rhythmic and synchronized epileptiform-like activity
Source: Sci Rep. 2021 May 31;11:11374. doi: 10.1038/s41598-021-90486-y (PMC8167095; doi:10.1038/s41598-021-90486-y)
Supplement: Supplementary file 1 — Supplementary Information 1. [file 41598_2021_90486_MOESM1_ESM.docx]

**Supplementary information**

## **Evaluation of neuronal death induced by SE-like activity**

Incubation of primary cultures of hippocampal or cortical neurons, or hippocampal slices, in a medium lacking Mg^2+^ ([Mg^2+^]_0_) is commonly used to model *Status Epilepticus in vitro* [^3^](#_ENREF_3)^,^[^47^](#_ENREF_47). Since this model is characterized by an excessive activity of excitatory synapses, here we first measured neuronal cell death induced by transient incubation of hippocampal neurons in a Mg^2+^-free solution.

Neuronal cell death caused by the experimental conditions used to model SE in vitro was assessed after incubation of cultured hippocampal neurons (15 DIV) with [Mg^2+^]_0_ medium for 30, 60, 90 and 120 minutes. After stimulation the [Mg^2+^]_0_ medium was replaced by culture-conditioned medium for an additional period of 8h (Post-incubation), and neuronal death was analysed via nuclear staining with Hoechst 33342. Exposure of cultured hippocampal neurons to epileptogenic induced a time-dependent cell death, as determined by analysis of nuclear morphology 8 h after the insult (Figure S1). The shorter period of incubation in [Mg^2+^]_0_ medium (30 min) did not induce cell death when compared with control conditions (incubation in control salt solution), while hippocampal neurons incubated for 60 or 90 min under the same conditions showed _~_15% increase in cell death, characterized by nuclear shrinking and chromatin condensation. Cell death was increased by _~_20% when the hippocampal neurons were exposed to [Mg^2+^]_0_ for 120 min (Figure S1).

Since we aim at investigating the alterations in neuronal activity following transient incubation of hippocampal neurons in [Mg^2+^]_0_ medium (to model SE), experimental conditions were chosen where cell death was not significantly increased when compared to control conditions. Therefore, 30 min was the maximum period of incubation in [Mg^2+^]_0_ medium used in all other experiments performed in this work to avoid artefacts resulting from excitotoxic injury, which is coupled to [Ca^2+^]_i_ dysregulation [^48^](#_ENREF_48).


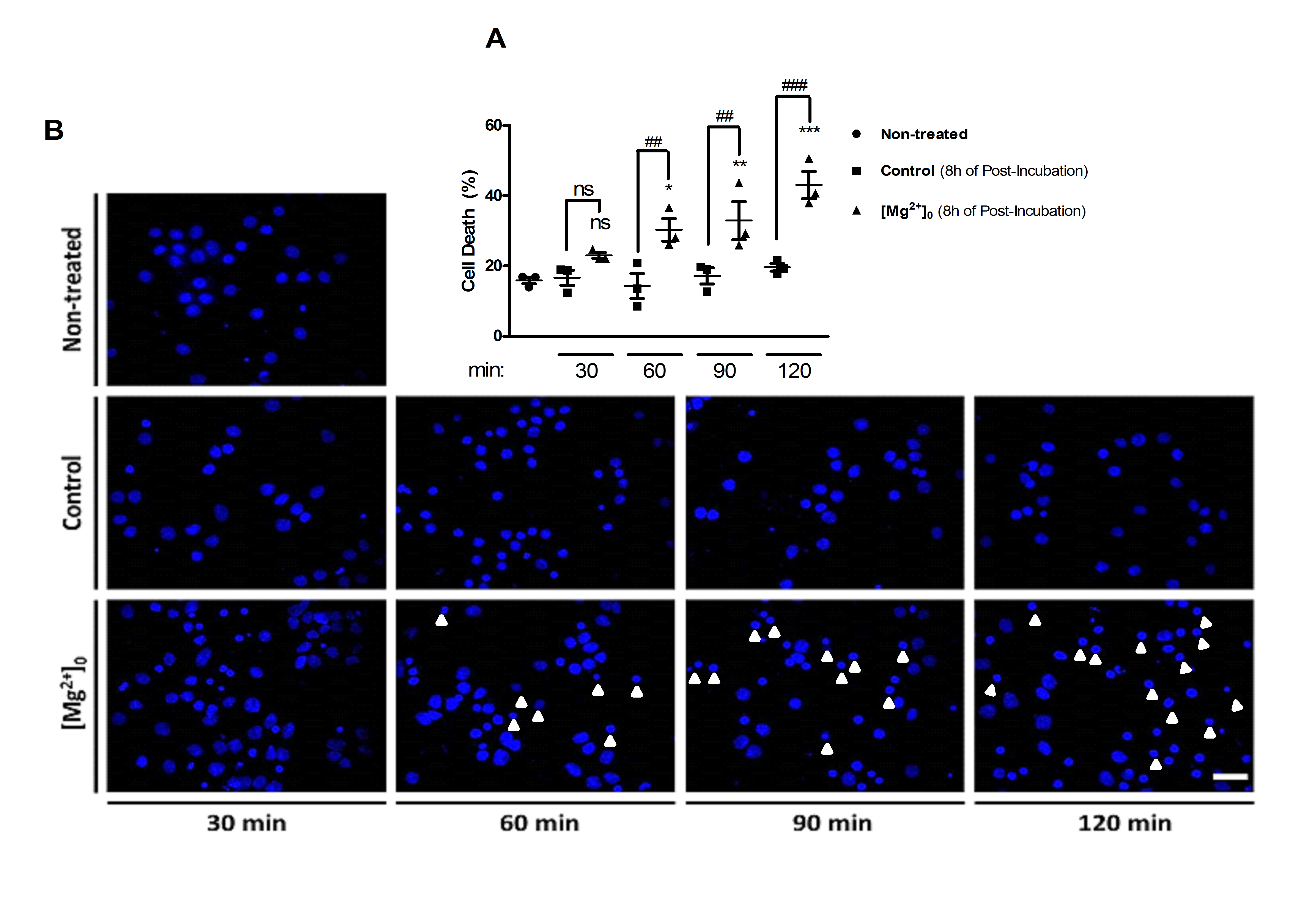


**Figure S1. Incubation of hippocampal neurons in [Mg^2+^]_0_ medium to model SE induces neuronal cell death in a time-dependent manner**. [A] Cultured hippocampal neurons (15 DIV) were incubated in [Mg^2+^]_0_ medium during 30, 60, 90 or 120 min. In control conditions, hippocampal neurons were incubated for the same period in a solution with the same composition but supplemented with 2 mM MgCl_2_. Immediately after stimulation the neurons were incubated in culture‐conditioned medium for 8 h (post‐incubation). Non-treated cells were maintained in cultured conditioned medium throughout the experiment. Cell death was analysed via nuclei staining with Hoechst 33342. [B] Representative results are shown in the lower part of the figure (scale bar 50 µm). The arrowheads point to apoptotic nuclei. The results are average ± SEM of 3 different experiments performed in duplicate and in independent preparations. Statistical analysis was performed by one‐way ANOVA, followed by Dunnett’s and Bonferroni's test. *p<0.05, **p<0.01, ***p<0.001, ^##^p<0.01, ^###^p<0.001; significantly different when compared to control conditions. Non-significant differences are indicated as ns.
